# Farinaceous Foods

**Published:** 1887-08-27

**Authors:** 


### Farinaceous Foods.

It is not easy to exaggerate the importance of the various kinds of foods. Now that the bulk of the population have deserted the country for the town, feeble appetites and defective digestions are increasingly common. This is especially the case with the weaker sex, whose indoor life tends to diminish that eagerness of desire for food which characterises the healthy stomach, and to make digestion a process of which they are too often disagreeably and even painfully conscious. How few inhabitants of large towns, who have reached the age of thirty, are ignorant of the pangs and gnawings of dyspepsia! So common indeed is this experience, that many people regard it as the normal condition of town-bred humanity. The food provided for us has something to do with this unpleasant result, as well as the want of pure air and exercise. The baker and the cook are highly important personages, much more so to most people than the Bishop or the Prime Minister. A Bishop may be unlawned and a Prime Minister impeached, but all that can be done to the cook is to dismiss her, with the prospect of jumping "out of the frying-pan into the fire" by getting one who is ten times worse.

If only we could buy food which could not be spoiled in the cooking, how blessed would be the condition of our households! Puddings and potatoes are the rocks on which many cooks split. But these constitute a very large proportion of our total food-stuffs. The man or woman who can guarantee the perfect potato every day for dinner is a treasure; and only second to them is the genius who can make milk puddings so palatable and digestible that neither ourselves nor our children will sigh after "roley-poley," "apple tart," or those enemies of bad consciences, "cabinet" or "plum." Mr. James Marshall, of Glasgow, has devoted study, time, and money to the preparation of a variety of foods from "the finest of the wheat." The results are eminently satisfactory. Samples of farola, tritola, granola, and other preparations have been sent to us, which we have tested in a variety of practical ways. These foods are all excellent. The original wheat from which they are prepared is manifestly of the very "finest"; and the methods adopted in the manipulation are such as to secure the most exact and various results. The farola, tritola, and granola are not only beautiful in appearance when uncooked, but can be made into a variety of the most ornamental and appetising dishes. The completed product, when the least care is exercised, is really delicious, and as digestible as it is pleasant in the eating. For ladies and children, and all of delicate digestion, there is nothing better in the market than Marshall's preparations. It is of importance to remember that they are as wholesome and nutritious as they are pleasant and appetising. If he who makes two blades of grass to grow where only one grew before, is a benefactor, so also is he who brings to the delicate and dyspeptic mortal something wherewith to please his fickle palate, and to persuade his stomach to do its duty promptly, effectively, and without pain.

